# Emergence of infectious malignant thrombocytopenia in Japanese macaques (*Macaca fuscata*) by SRV-4 after transmission to a novel host

**DOI:** 10.1038/srep08850

**Published:** 2015-03-06

**Authors:** Munehiro Okamoto, Takayuki Miyazawa, Shigeru Morikawa, Fumiko Ono, Shota Nakamura, Eiji Sato, Tomoyuki Yoshida, Rokusuke Yoshikawa, Kouji Sakai, Tetsuya Mizutani, Noriyo Nagata, Jun-ichiro Takano, Sachi Okabayashi, Masataka Hamano, Koji Fujimoto, Takaaki Nakaya, Tetsuya Iida, Toshihiro Horii, Takako Miyabe-Nishiwaki, Akino Watanabe, Akihisa Kaneko, Akatsuki Saito, Atsushi Matsui, Toshiyuki Hayakawa, Juri Suzuki, Hirofumi Akari, Tetsuro Matsuzawa, Hirohisa Hirai

**Affiliations:** 1 Center for Human Evolution Modeling Research, Primate Research Institute, Kyoto University, Inuyama, Aichi 484-8506, Japan; 2 Laboratory of Signal Transduction, Department of Cell Biology, Institute for Virus Research, Kyoto University, Kyoto 606-8507, Japan; 3 National Institute of Infectious Diseases, Toyama 1-23-1, Shinjuku-ku, Tokyo 162-8640, Japan; 4 The Corporation for Production and Research of Laboratory Primates, 1-16-2 Sakura, Tsukuba, Ibaraki 305-0003, Japan; 5 Department of Infection Metagenomics, Genome Information Research Center, Research Institute for Microbial Diseases, Osaka University, 3-1 Yamadaoka, Suita, Osaka 565-0871, Japan; 6 Tsukuba Primate Research Center, National Institute of Biomedical Innovation, 1-1 Hachimandai, Tsukuba, Ibaraki 305-0843, Japan; 7 Department of Infectious Diseases, Kyoto Prefectural University of Medicine, 465 Kawaramachi-hirokoji, Kamigyo-ku, Kyoto, Japan; 8 Department of Molecular Protozoology, Research Institute for Microbial Diseases, Osaka University, Suita, Osaka, Japan; 9 Department of Brain and Behavioral Sciences, Primate Research Institute, Kyoto University, Inuyama, Aichi 484-8506, Japan; 10 Department of Molecular Biology, Primate Research Institute, Kyoto University, Inuyama, Aichi 484-8506, Japan

## Abstract

We discovered a lethal hemorrhagic syndrome arising from severe thrombocytopenia in Japanese macaques kept at the Primate Research Institute, Kyoto University. Extensive investigation identified that simian retrovirus type 4 (SRV-4) was the causative agent of the disease. SRV-4 had previously been isolated only from cynomolgus macaques in which it is usually asymptomatic. We consider that the SRV-4 crossed the so-called species barrier between cynomolgus and Japanese macaques, leading to extremely severe acute symptoms in the latter. Infectious agents that cross the species barrier occasionally amplify in virulence, which is not observed in the original hosts. In such cases, the new hosts are usually distantly related to the original hosts. However, Japanese macaques are closely related to cynomolgus macaques, and can even hybridize when given the opportunity. This lethal outbreak of a novel pathogen in Japanese macaques highlights the need to modify our expectations about virulence with regards crossing species barriers.

The Japanese macaque (*Macaca fuscata*), also known as the snow monkey, is a species of primate indigenous to the Japanese archipelago making it the most northern-living extant non-human primate (NHP). Because of their amenable behavioral and cognitive characteristics, Japanese macaques are highly suitable for use as experimental animals in research, particularly in brain sciences^1^. Over 800 Japanese macaques are currently maintained and bred at the Primate Research Institute, Kyoto University (KUPRI).

Recently, a hemorrhagic syndrome arising from thrombocytopenia of unknown origin affected the Japanese macaques kept at KUPRI, many of which died as a result, an event that was reported as a mysterious illness in Nature News^2^. This disease was first observed in two macaques in July 2001 and in five more in 2002, after which six of these macaques died (phase 1). After the first outbreak ended, there were no further incidences of Japanese macaques exhibiting hemorrhagic syndrome for the following six years. However, a second outbreak (phase 2) with the same symptomology occurred in March 2008, with 43 macaques contracting the disease between then and February 2011; 42 of these macaques died (including those euthanized because of poor prognosis). Macaques that contracted the disease exhibited anorexia, lethargy, pallor, nasal hemorrhage, gingival hemorrhage, petechiae, ecchymoses, and melena. After the onset of symptoms, the fatality rate was extremely high and only one macaque survived in each outbreak. Severely decreased blood platelet (PLT) counts and lowered white blood cell (WBC) and red blood cell (RBC) counts were found in all macaques that developed the disease, with PLT counts close to zero at the time of death in nearly all cases. All macaques were active as usual even on the day prior to the onset, with almost all eating normally and showing no early signs of disease. Even veteran caretakers were unable to foresee the onset based on the general conditions of the macaques. The onset was very sudden and death occurred within a very short period of time; thus, little could be done to save or treat the affected macaques. Such peracute thrombocytopenia has not occurred in any primates other than Japanese macaques and there have been no reports worldwide of this disease.

Since the first epidemic, researchers at KUPRI have attempted to determine its cause. The situation was briefly introduced in a report written in Japanese (Kyoto University Primate Research Institute Disease Control Committee 2010); however, the etiology was still unclear when the report was submitted. As written in the news in *Nature* in 2010, we considered that the illness was probably not due to any known agents, including those such as Ebola, that induce hemorrhagic fevers^2^.

During phase 2, we organized a collaborative team with other institutions to investigate the disease. The causative agent of this unique disease in Japanese macaques was investigated by five research institutions (KUPRI; Institute for Virus Research, Kyoto University; National Institute of Infectious Diseases, Japan; the Corporation for Production and Research of Laboratory Primates; Research Institute for Microbial Diseases, Osaka University) using different research techniques and complementary approaches. This multilateral study allowed us to conclude that the thrombocytopenia in Japanese macaques was caused by infection with simian retrovirus type 4 (SRV-4), which we suspect originated via cross-infection from cynomolgus macaques.

## Results

### Thrombocytopenia in Japanese macaques

Table 1 shows the individual numbers of animals that developed the disease in the first and second outbreaks, along with details regarding sex, age, date of onset, date of death, and blood data. Japanese macaques at KUPRI were kept separately according to where they originated and the letters (e.g., TH, AR, and HG) before each individual number represent the birthplace of the animal or its ancestors. Onset was determined on the basis of decreased PLT counts or the pathological findings at necropsy. The disease progressed extremely rapidly after the onset (death within zero to a few days), and in some macaques, the disease was detected only after death; thus, blood data were not available for these individuals. Severely decreased PLT counts and lowered WBC and RBC counts were found in all macaques that developed the disease, with PLT counts close to zero at the time of death in nearly all cases. Blood data from healthy Japanese macaques kept at KUPRI showed that the normal levels were PLT counts of 29.0 ± 7.8 × 10^4^/μl, WBC counts of 12.2 ± 3.8 × 10^2^/μl, and RBC counts of 505 ± 37 × 10^4^/μl. These data indicated that the macaques that contracted the disease had extreme anemia. The Japanese macaques that contracted the disease did not share any attributes such as sex, birthplace, age, or genealogy. Life prolongation was possible to some extent with blood transfusion, but treatments with any drugs or antibiotics had no effect at all. As a result, many diseased macaques were euthanized from November 2009 onward, and samples were collected from them. After the onset occurred, the fatality rate was extremely high and only one macaque survived each outbreak. PLT counts recovered in these two macaques.

### Clinical investigation and necropsy

**Figure 1:**
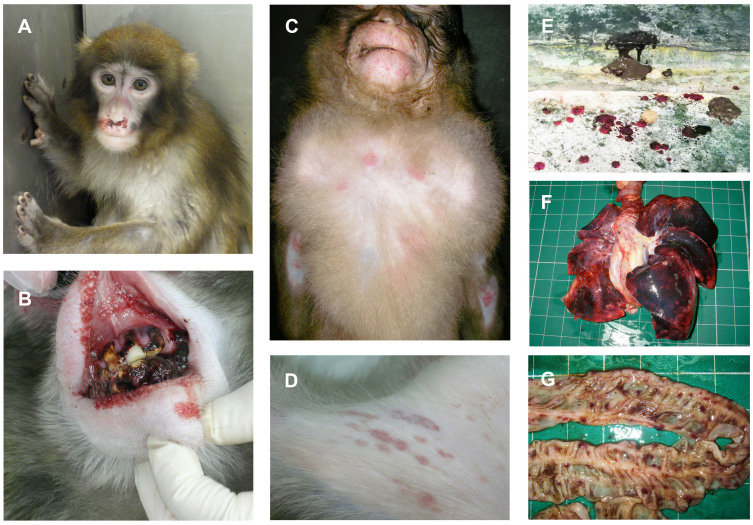
Clinical symptoms found in Japanese macaques that developed severe thrombocytopenia. The main clinical findings in the macaques that contracted the disease were reduced appetite, recumbency, facial pallor, hemorrhaging of the nasal mucosa and gums, subcutaneous bleeding, and brown-colored mucous and bloody stool. Necropsy of the dead macaques revealed hemorrhaging of all tissues, with particularly marked petechial hemorrhaging of the serosa and/or the mucous surface of the digestive tract and pulmonary hemorrhaging. (A) Facial pallor and hemorrhaging of the nasal mucosa, (B) bleeding from the alveolar ridge, (C) subcutaneous hemorrhage (chest), (D) subcutaneous hemorrhage (groin), (E) brown-colored mucous and bloody stool, (F) diffuse hemorrhaging of the lung, and (G) petechiae of the intestine. Photo courtesy of M.O., J.S., T.M.-N., A.W. and A.K.

The main clinical findings in the macaques that contracted the disease were reduced appetite, recumbency, facial pallor, hemorrhaging of the nasal mucosa and gums, subcutaneous bleeding, and brown-colored mucous and bloody stool (external appearance; Fig. 1A–E). Animals that exhibited these symptoms died within one to three days. Necropsy of the dead macaques revealed hemorrhaging of all tissues, with particularly marked petechial hemorrhaging of the serosa and/or the mucous surface of the digestive tract, and pulmonary hemorrhaging (necropsy findings; Fig. 1F, G). Splenomegaly was observed in some macaques, but other macroscopic lesions could not be identified.

### Blood examination

**Figure 2:**
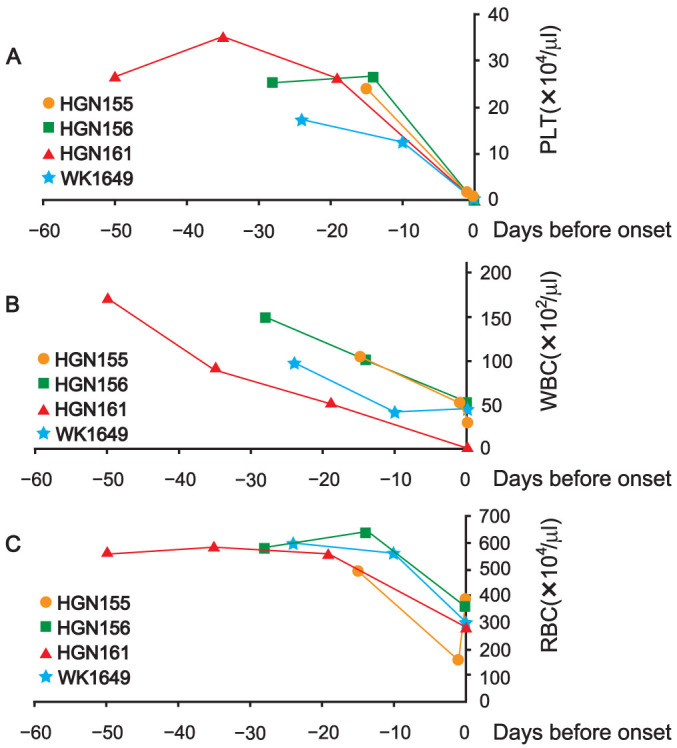
Numbers of platelets (A), leukocytes (B), and erythrocytes (C) in Japanese macaques that developed severe thrombocytopenia. PLT counts were maintained at 10 × 10^4^/μl or above from two weeks to ten days before the onset, after which they dropped rapidly. Furthermore, PLT and WBC counts dropped before RBC counts decreased.

Figure 2 shows the changes in PLT, WBC, and RBC counts of the affected macaques. Because the onset was sudden, as stated above, little information from prior to the onset was available. However, blood data were monitored in some macaques (ID: HGN155, HGN156, HGN161 and WK1649) that were kept in the same breeding rooms as the macaques that contracted the disease. These data showed that PLT counts were maintained at 10 × 10^4^/μl or above from two weeks to ten days before the onset, after which they dropped rapidly. Furthermore, PLT and WBC counts dropped before RBC counts decreased. However, no remarkable changes were noted in any other serum biochemical values, and the levels of C-reactive protein (CRP), a marker of inflammation, did not increase in any macaque that contracted the disease (data not shown).

### Antibody tests against viral pathogens

Antibodies against Ebola, Marburg, Lassa, and Crimean–Congo hemorrhagic fever viruses, which induce hemorrhagic syndromes in humans, were all negative. Neutralizing antibodies against canine distemper virus (CDV) were also negative. The results of the antibody tests against eight simian pathogenic viruses are shown in Table 2. Antibody prevalence levels against simian Epstein–Barr virus (SEBV), cytomegalovirus (CMV), and simian foamy virus (SFV) were quite high in the affected macaques; however, the levels in the healthy controls were also high. Thus, there appeared to be no correlations between the disease and these viruses. Anti-SRV antibodies were negative in all affected macaques tested.

### Electron microscopy (EM)

**Figure 3:**
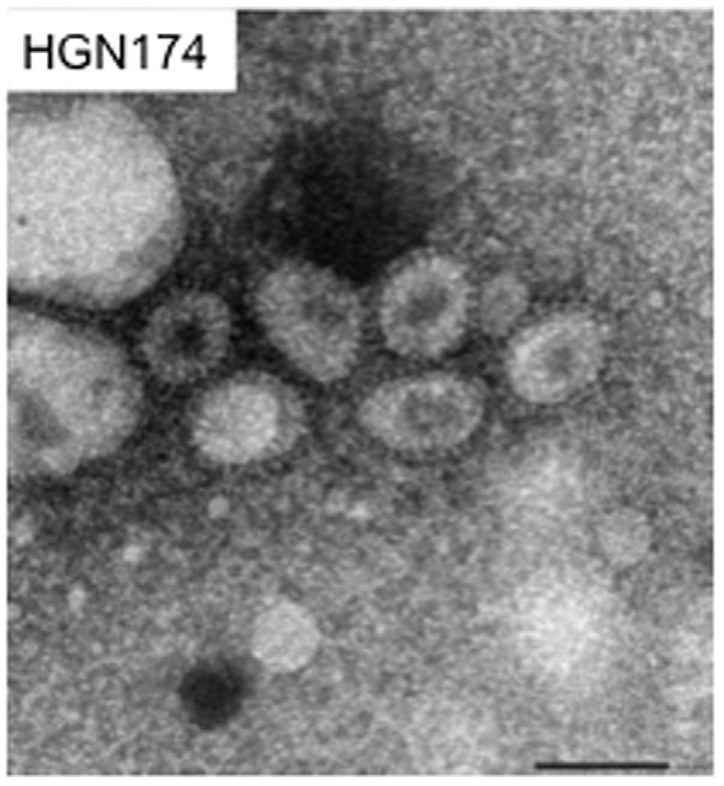
Electron microscopy. Numerous viral particles were observed in the plasma of Japanese macaques that developed thrombocytopenia. Viral particles had prominent spikes and measured 70–120 nm in diameter. Bar indicates 100 nm.

EM analysis detected the presence of enveloped viral particles with prominent spikes, which measured 70–120 nm in diameter, in the plasma of 3/3 Japanese macaques (ID: HGN174, HGN181 and IZ1470) showing symptoms (Fig. 3).

### PCR and RT-PCR analyses of viral pathogens

All DNA viruses listed in Table 3 were not detected by the specific PCR in the plasma of 3/3 Japanese macaques (ID: HGN174, HGN181 and IZ1470) that contracted severe thrombocytopenia. In the case of RNA viruses, SRV and SRV-4 (Table 3, 12 and 13) but none of the other RNA viruses were positive according to RT-PCR (data not shown).

### Rapid determination of viral sequences (RDV) method

RDV was performed to examine the affected macaques (ID: HGN174, HGN181 and IZ1470), and we determined the nucleotide sequences of 136, 118, and 50 amplicons from RNA viruses, double-stranded (ds) DNA viruses, and single-strand (ss) DNA viruses, respectively. However, we did not detect any known ds DNA or ss DNA viral sequences, whereas we detected partial sequences that were virtually identical to SRV-4 in 4/136 amplicons from RNA viruses.

### Metagenomic analysis

To further explore the existence of pathogens, we independently performed metagenomic analysis of the plasma of an affected macaque (ID: TBN201). Metagenomic shotgun sequencing using 454 GS Junior yielded 61,986 sequence reads in total. We detected 485 sequence reads, which provided widespread coverage of the genome of SRV-4 with high homology (Supplementary Fig. S1). Other candidate viral genomes were not detected by metagenomic analysis.

### Virus isolation

Virus isolation tests using Vero E6, human SLAM-Vero, canine SLAM-Vero cells, and embryonated egg cultures were performed using plasma samples, which detected no cytopathic effect (CPE) in the cell cultures after five serial passages, and no abnormalities were observed in egg cultures after five days of incubation. The inoculated Vero cells and human and canine SLAM-expressing Vero cells were examined for the presence of viral antigens using the plasma of affected macaques. However, no viral antigens were detected in the cells by indirect immunofluorescence tests (data not shown). A hemagglutination test using the allantoic fluid of the inoculated eggs and RBCs of turkeys was also negative (data not shown).

When peripheral blood mononuclear cells (PBMCs) from the SRV-4-provirus positive Japanese macaques (ID: TH1626 and IZ1470) were cocultivated with Raji cells, typical SRV CPE including syncytia were observed after three days of cocultivation. DNA from Raji cells were examined by nested PCR using SRV-4 specific primers, and PCR products of the expected size were observed following agarose gel electrophoresis (data not shown).

### Detection of SRV-4 viral genome by RT-PCR or PCR

**Figure 4:**
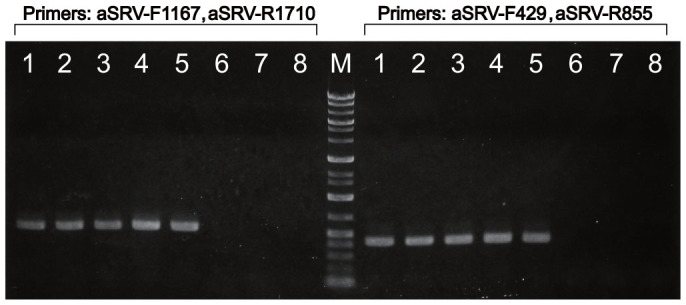
Detection of SRV-4 viral RNA by RT-PCR. Plasma of five affected macaques (lane: 1–5) were examined by RT-PCR using the primer set, aSRV-F1167 and aSRV-R1710. To confirm the results, same samples were re-tested using another primer set, aSRV-F429, aSRV-R855. Each primer sets amplified a single expected products respectively. In contrast to the affected macaques, two healthy controls reared at the second campus (lane: 6–7) were negative. 1: WK1655 (2008/3/12), 2: TH2158 (2008/3/15), 3: AR1694 (2008/5/15), 4: AR1995 (2008/5/23), 5: TH2041 (2009/6/22). 6: OKN244 (2010/12/14), 7: OKN245 (2010/12/14) 8: H_2_O. Figures in each parenthesis represent the date of blood collection. Blood from each affected macaques was collected just after onset of thrombocytopenia. Photo courtesy of M.O.

RT-PCR using each of two primer sets for viral RNA from affected macaques amplified single products of the expected size. Thirty affected macaques for which sera or plasma were stored in KUPURI, including the 14 macaques, which were examined by antibody tests (Table 2), were all positive for SRV-4 in both RT-PCR test. In contrast, healthy controls at the second campus of KUPRI were all negative for SRV. Partial results of RT-PCR were shown in Fig. 4.

Nested PCR for proviral DNA revealed that the 14 affected macaques described above were all positive for SRV-4. On the other hand, healthy controls at the second campus were all negative (data not shown).

### Phylogenetic analyses

After RT-PCR and sequencing using SRV genomic RNA from one affected macaque (ID: OK2015), a partial sequence of *gag* gene (514 bp) was determined. The nucleotide sequence data obtained in this study is available in the DDBJ/EMBL/GenBank databases under the accession number AB933257. The same sequence was also detected in viral RNA extracted from affected macaques (ID: IZ1470, TH1626, TBN201, TH2158 and AR1649) (data not shown).

**Figure 5:**
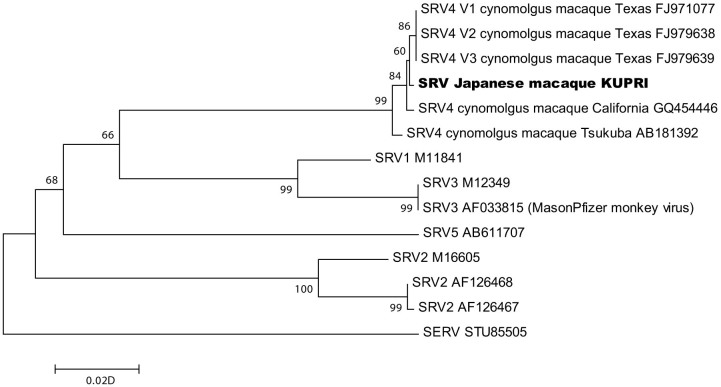
Phylogenetic relationships among simian retroviruses and SRV-4 detected in Japanese macaques at KUPRI. A phylogenetic tree was constructed from the partial *gag* sequences (514 bp) using the neighbor-joining method. The phylogenetic tree was evaluated using a bootstrap test based on 1,000 resamplings. The sequence of simian endogenous retrovirus was used as an outgroup to indicate the location of the root of the ingroup. The tree indicates that the SRV from Japanese macaques clustered with the SRV-4 strains isolated in Texas and California, USA, and Tsukuba, Japan. Numbers on the nodes represent bootstrap values. Scale bar represents the evolutionary distances.

Figure 5 shows the phylogenetic relationships among SRVs, which were inferred from the partial *gag* sequences. The phylogenetic tree indicates that the SRV detected here from Japanese macaques clustered with the SRV-4 strains isolated from cynomolgus macaques in Texas and California, USA^4^, and Tsukuba, Japan^5^.

## Discussion

The disease described here was first detected in 2001 in two female Japanese macaques (one in July and one in August) housed in the same cage complex (but in different individual cages). Both macaques died two days after the first examination by veterinarians. The progress of the disease was peracute in all macaques that subsequently contracted it. Excluding a few cases, all macaques exhibited systemic hemorrhagic symptoms such as those shown in Fig. 1, and they died within a few days of onset (Table 1). Only two macaques survived the two outbreaks. At the time of the first examination of the affected macaques by veterinarians, i.e., when their responsible caretakers first noticed clinical symptoms such as subcutaneous hemorrhaging or mucous and bloody stool, their PLT, WBC, and RBC counts had already dropped markedly and they had severe anemia (Table 1). Since PLT counts had dropped to below 1 × 10^4^/μl, the clinical findings of subcutaneous hemorrhage, mucous and bloody stool, and gingival hemorrhage were considered to be consequences of their decreased PLT counts. On the other hand, five macaques (ID: AR2182, TH1439, HGN160, HGN267 and HGN165) that were kept in the same room as the affected macaques were euthanized when their PLT counts dropped below 10 × 10^4^/μl, because we predicted that they had a poor prognosis. However, four of these macaques maintained their PLT counts to some degree at the time of euthanasia, and necropsy showed that the hemorrhage lesions were mild. It is possible that these macaques may not have progressed to onset, i.e. PLT counts may not have become near zero. Therefore it is doubtful whether these macaques should be included in the same category as the severely affected macaques. Because this is the first report of infectious thrombocytopenia in Japanese macaques, all data from the macaques that possibly developed the disease are shown in Table 1, but further investigations are needed to determine specific criteria for onset.

As shown in Fig. 2, PLT counts were maintained at over 10 × 10^4^/μl from two weeks to ten days before the onset but dropped markedly thereafter. We also found that PLT and WBC counts decreased before RBC counts decreased. Considering the life span of each type of blood cell, it is possible that acute bone marrow dysfunction occurred within one month of the onset. Detailed pathological analysis is currently ongoing, although marked decreases in bone marrow cells have been confirmed by bone marrow smear examination (data not shown).

Despite not knowing the causative agent at the time, we suspected viral infection during each outbreak because C-reactive protein (CRP), which typically increases when inflammation occurs, did not increase in any of the affected macaques, while WBC counts decreased and treatments with antibiotics were completely ineffective. The clinical symptoms reminded us of hemorrhagic fever caused by viruses such as Ebola^6^, thus, we first investigated antibodies for several hemorrhagic fever viruses, which can affect other animals including humans. However, all macaques were negative for the antibodies against those pathogens. The antibody tests for eight macaque pathogens indicated no clear correlations between the onset and control groups, and no cause-and-effect relationships could be demonstrated for any of the pathogens. All affected macaques were negative for the anti-SRV antibody. These same examinations were also performed on the seven macaques affected in the first outbreak, all of which tested negative for the anti-SRV antibody. It has been reported that anti-SRV antibodies may not have been produced in a small subset of cynomolgus macaques infected with SRV^7^. we could not assume that no affected Japanese macaques had produced such antibodies at all, and thus, we excluded SRV as a causative virus. Therefore, we considered that it was highly likely that this disease was caused by an unknown pathogen^2^.

We tried to preserve samples from affected monkeys as much as possible, but most of samples were fixed with formalin and raw or frozen samples were quite limited. Therefore we handed the samples, which had been stored relatively in the large amount, to the several organizations and carried out the different examination at each organization. Although the results that each examination provided were not necessarily enough, we obtained the evidence suggesting a close association between SRV-4 and thrombocytopenia in the Japanese macaques. Our investigations demonstrated as follows: Large numbers of viruses with retrovirus-like morphology were present in the plasma of the Japanese macaques that developed thrombocytopenia. In addition, SRV-4 genomes were confirmed in the plasma of all affected macaques and SRV-4 was isolated from PBMCs at the Corporation for Production and Research of Laboratory Primates. Later, SRV-4 was also isolated from the plasma, feces, and bone marrow cells of a Japanese macaque that exhibited severe thrombocytopenia at the Institute for Virus Research, Kyoto University (unpublished data). In contrast, SRV-4 genomes were not detected in macaques that were raised at the second campus and who had no contact at all with the affected macaques. Furthermore, no candidate pathogens other than SRV-4 could be detected in the affected macaques despite the use of the RDV method, electron microscopy and virus isolation. Since the RDV method is used to comprehensively detect viral genomes, it is extremely significant that RDV could not detect any other candidates. Metagenomic analysis for RNA also only detected SRV-4 in the affected macaque (ID: TBN201), which was not used for above-mentioned examinations. This fact strongly supports that SRV-4 is the causative agent of thrombocytopenia in Japanese macaques. Additional research is required to determine whether this disease occurs with SRV-4 alone or whether some co-factors are associated with thrombocytopenia; however, it is clear that SRV-4 was the principal agent responsible for this disease.

SRV is classified in the *Betaretrovirus* genus and both endogenous and exogenous variants have been identified in NHPs^4^. Seven serotypes of exogenous SRV have been identified and six of these are known to infect at least eight species of macaques^8^,^9^. However, no SRV serotypes have been reported in wild Japanese macaques. SRV-4 has been found only in cynomolgus macaques kept in experimental animal facilities^4^,^10^,^11^, e.g. at the Tsukuba Primate Research Center in Japan^5^,^7^,^12^,^13^. Various species of macaques, including cynomolgus macaques, have been kept at KUPRI since their introduction from other facilities; thus, we assume that these translocated macaques brought SRV-4 to KUPRI. More than one species of macaque has sometimes been kept in the same room depending on the research purpose, and injured or diseased macaques may have been hospitalized in the same room for medical treatments, although they were kept in different cages. It is likely that SRV-4 was transmitted from a carrier cynomolgus macaque to a Japanese macaque in the past, and that only Japanese macaques developed severe thrombocytopenia as a consequence.

SRV has been identified as the etiology of an infectious immunosuppressive syndrome, so-called Simian AIDS, in several species of macaques at primate research centers in USA such as the Washington National Primate Research Center and the California National Primate Research Center^8^. It was also reported that SRV-induced immunosupression brought on neoplastic diseases in infected macaques. For instance, SRV-2 is specifically associated with a proliferative condition called retroperitoneal fibromatosis (RF) of infected macaques, including pigtailed macaques, crab-eating macaque, rhesus macaques, and Celebes macaques. In these, pigtailed macaques are most susceptible and their clinical symptoms are severe. It is suspected that RF is due to a gammaherpesvirus acting as cofactors secondary to SRV infection and immune suppression. Recently pathogenicities of SRV-4 have been reported^4^,^5^,^13^. SRV-4 is usually asymptomatic when it infects cynomolgus macaques, but may cause immunosuppression marked by chronic diarrhea and some macaques may exhibit mild thrombocytopenia. However, thrombocytopenia in cynomolgus macaques is not inevitable and the disease can also progress chronically. In this way, no cases of the peracute thrombocytopenia seen in the Japanese macaques have been reported. We consider that the virus in the cynomolgus macaque crossed the so-called species barrier and infected and spread to Japanese macaques, causing severe thrombocytopenia. It is a well-known fact that crossing the species barrier increases pathogen virulence^14^. Viruses such as the avian influenza virus^15^ and the severe acute respiratory syndrome (SARS) virus^16^ exhibited strong pathogenicity after crossing the species barrier from their natural hosts to humans. In NHPs, herpesvirus saimiri can spread from naturally infected squirrel monkeys to other new world monkeys, causing serious lymphoma^17^. After crossing the species barrier into new hosts, the virus exhibits unexpectedly severe symptoms that are not observed in the original hosts. In most cases, the new hosts are distantly related to the original host^15^,^16^. In the case of SRV-4, however, although the virus spread from cynomolgus macaques to Japanese macaques, which are closely related and both belong to the *fascicularis* group of the genus *Macaca* (hybridization may also occur between the two species), extremely severe acute symptoms developed. This disease merits attention because it has changed our concept of the relationship between species barriers, genetic distance and virulence.

Despite the causative pathogen being SRV, a known virus, its identification took over ten years from the first outbreak. Nearly 30 years have passed since the discovery of the human immunodeficiency virus (HIV), which causes acquired immunodeficiency syndrome (AIDS), and the development of an animal model was urgently required at the time of its discovery^18^,^19^. As mentioned above, SRV causes immunosuppression in infected macaques; thus, it was used as a model of AIDS^20^,^21^. Indeed, a large amount of research was conducted using SRV from the mid-1980s to the 1990s, and many reports have been published^8^,^22^. Serotypes 1 to 5 were also discovered at an early stage^23^. However, after it was discovered that HIV was the result of a cross-species transmission of simian immunodeficiency virus (SIV)^24^,^25^, interests of researchers were immediately shifted from SRV to SIV, and a few reports on SRV have been published recently. SRV is a relatively small RNA virus with a full length of just over 8,000 base pairs, but the full sequence of the SRV-4 genome was only reported in 2010^4^. Thus, the reason why we required a long period of time to detect the cause of the disease was not only the fact that the affected macaques did not produce antibodies but also because an effective molecular diagnosis method had not been developed.

Furthermore, the severe symptoms in the Japanese macaques were very different from those that were known to be caused by SRV in other hosts. Thus, we have held the biased view that “SRV could not cause such a severe disease.” Many cynomolgus macaques are imported into Japan every year for experimental purposes; however, SRV is not included among the items in the imported quarantine inspection and few facilities can screen for it independently. In animal facilities in Japan, several species of macaques are often kept in the same room because of lack of space and other reasons. Moreover, Japanese macaques and cynomolgus macaques are also often kept in adjacent cages in zoos in Japan. Nevertheless, similar disease outbreaks have not been reported at other facilities. These facts, as well as the severe symptoms, led us to believe that an unknown pathogen was involved. The article that introduced this disease in *Nature* mentioned that, “Because Japanese laboratories tend to have excellent diagnostic capabilities, the illness is probably not due to any of the known agents”^2^. We still have reservations about why this thrombocytopenia occurred only at KUPRI and whether SRV-4 alone can actually cause this disease. In light of this, it may be said that the metagenomic analysis and the RDV method, which objectively detected the viral genome, are epoch-making diagnostic techniques.

According to Koch's postulates, the final identification of a pathogen requires experimental infection of the hosts. However, experimental infection would be dangerous in this case because the symptoms of this disease are extremely severe and no treatment has been established. More than eight hundreds of Japanese macaques are reared in KUPRI, but we don't have a suitable facility for experimental infection of viral pathogens in KUPURI. Therefore, our colleagues in Institute for Virus Research, Kyoto University, have performed experimental infections at the P3A level animal facility in their Institute. SRV-4 from one of the affected Japanese macaques as well as an infectious molecular clone derived from isolated SRV-4 were inoculated into Japanese macaques. As a result, the isolate induced severe thrombocytopenia in all four macaques within 37 days. Infectious molecular clone-derived virus could also develop same symptoms (unpublished data).

This disease is a great threat against the Japanese macaque, a primate that is indigenous to Japan, and its spread to the natural population must be prevented. The pathology, mechanism of onset, and natural host of this disease must be clarified. In addition, the establishment of the diagnostic method and construction of the quarantine inspection system should be done urgently. ELISA is usually a simple, easy and extremely effective diagnostic method for infectious disease. However it is revealed that no specific antibody against SRV-4 was detected in affected macaques. Therefore molecular diagnosis for SRV, such as PCR for proviral DNA or RT-PCR for viral genome, is effective. The pathogenicity for Japanese macaques of SRV-4 variants or other serotypes is still unclear, but the molecular diagnostic methods that can detect them will be necessary. As described above, Japan is importing more than 5000 cynomolgus macaques every year from Southeast Asia where the prevalence of SRV may be quite high. However SRV is not included among the items in the imported quarantine inspection. Unrecognized co-infection with SRV in the biomedical model may severely compromise the integrity of toxicology studies. SRV infection is a threat not only against the Japanese macaque but also against biomedical researches. Importers and researchers using cynomolgus macaques should carry out inspection of SRV absolutely. In future, some kind of legal regulation may be necessary.

We had not carried out special rearing management before we suspected that the cause of illness was an infectious disease. Even so, the humans who handled the macaques, including researchers, veterinarians and caretakers, showed no symptoms of thrombocytopenia. There is no report on SRV-associated thrombocytopenia of humans even from Southeast Asia, where wild macaques are naturally infected with SRV and humans may come into close contact with those macaques regularly. Therefore SRV-4 is less likely to cause thrombocytopenia in humans. In this study, however, we isolated SRV-4 using the Raji cell line, which is the continuous human cell line from hematopoietic origin. It seems premature to conclude that humans are not susceptible to SRV-4 as cautioned by Cyranoski^2^.

## Methods

### Primate Research Institute, Kyoto University (KUPRI)

KUPRI houses 13 species (*Pan troglodytes*, *Hylobates agilis*, *Papio hamadryas*, *Macaca fuscata*, *Macaca mulatta*, *Macaca cyclopis*, *Macaca fascicularis*, *Macaca radiata*, *Ateles belzebuth*, *Cebus apella*, *Aotus trivirgatus*, *Saguinus oedipus*, *Callithrix jacchus*) and ca. 1,200 individual nonhuman primates including 800 Japanese macaques, 250 rhesus macaques, 140 common marmosets, 13 chimpanzees, and 11 cynomolgus macaques. The macaques are kept either in individual primate cages (W900 × D650 × H820 mm) in air-conditioned rooms, in outdoor group cages or in open enclosures. Pelleted diets (AS, Oriental Yeast Co., Ltd. Tokyo, Japan) and supplementary diets such as fruit and vegetables are provided twice a day. Fresh water is provided by an automatic watering system. At KUPRI, environmental enrichment has been promoted to improve behavior and psychological conditions of all macaques.

### Japanese macaques

We examined affected Japanese macaques that were dead or euthanized. As non-infected controls, we also examined Japanese macaques that were raised at the second campus at KUPRI, among which there were no affected macaques.

### Ethics statement

The care and use of Japanese macaques adhered to the Guidelines for Care and Use of Nonhuman Primates (Version3) by the “Animal Welfare and Animal Care Committee (Monkey Committee)” of KUPRI. These guidelines were prepared based on the provisions of the Guidelines for Proper Conduct of Animal Experiments (June 1, 2006; Science Council of Japan), Basic Policies for the Conduct of Animal Experiments in Research Institutions under the Jurisdiction of the Ministry of Health, Labor and Welfare [effective on June 1, 2006; Ministry of Health, Labor and Welfare (MHLW)], Fundamental Guidelines for Proper Conduct of Animal Experiment and Related Activities in Academic Research Institutions [Notice No. 71 of the Ministry of Education, Culture, Sports, Science and Technology (MEXT) dated June 1, 2006] and Standards Relating to the Care and Management of Laboratory Animals and Relief of Pain (Notice No. 88 of the Ministry of the Environment dated April 28, 2006). This study was carried out in accordance with the approved guidelines.

No specific animal research protocol was drafted for this study as only clinical samples were analyzed for diagnostic purposes. The protocol to collect samples in control macaques was reviewed and approved by the Monkey Committee at KUPRI, and then authorized by the Kyoto University Animal Experimentation Committee (2011-115). Extensive veterinary care was provided to all macaques affected to minimize pain and distress. Macaques in extreme thrombocytopenia were humanely euthanized using overdose of pentobarbital by veterinarians.

### Clinical investigation and necropsy

Clinical investigations were performed on affected macaques and some macaques who were kept in the same room together with the affected macaques. Symptomatic treatment were initially provided to the affected macaques, however these treatments were not effective. After Nov. 2009, the affected macaques were euthanized due to poor prognosis and risk of spreading infection. Necropsies were performed on all dead and euthanized macaques, and samples were taken for further investigation.

### Antibody tests against viral pathogens

To determine the virus that was responsible for the disease, antibodies against Ebola, Marburg, Lassa, and Crimean–Congo hemorrhagic fever viruses, all of which induce hemorrhagic syndromes in humans, were examined at the National Institute of Infectious Diseases, Japan. Neutralizing antibodies against CDV were also tested, because CDV is known to cause a lethal disease in macaques^26^.

Antibody tests against eight primate viral agents [SEBV, CMV, simian varicella virus (SVV), B virus (BV), SIV, simian T-lymphotropic virus (STLV), SFV, and SRV] were conducted at the Corporation for Production and Research of Laboratory Primates. The corporation is an inspection agency which established original testing procedures for monkey viruses. Detailed procedures for the antibody tests used at the corporation have not been published. However, the corporation is the only inspection agency in Japan that can perform these antibody tests. Most primate research institutes in Japan use this inspection agency and it is trusted.

### Electron microscopy (EM)

Viral particles in the plasma of three Japanese macaques that developed thrombocytopenia were examined by transmission EM. The plasma was diluted three times with phosphate-buffered saline (PBS) and centrifuged at 3,000 rpm for 20 min. The supernatants were clarified further by centrifugation at 8,000 rpm for 30 min. The virus particles, if any, were pelleted using 20% (w/v) sucrose in PBS at 31,000 rpm for 2 h, resuspended in PBS, and subjected to EM analysis. The samples were fixed with 4% glutaraldehyde, negatively stained with 2% phosphotungstic acid, and observed under a JEM-1400 transmission electron microscope (JEOL Ltd., Tokyo, Japan).

### Isolation of viral RNA and proviral DNA

Viral RNA was isolated from the plasma or serum of macaques at onset using a QIAamp Viral RNA Mini Kit (Qiagen, Tokyo, Japan) or a High Pure Viral RNA Kit (Roche Diagnostics, Tokyo, Japan). Proviral DNA was also isolated from blood using a DNeasy Blood & Tissue Kit (Qiagen).

### RT-PCR and PCR analyses of viral pathogens

Viral pathogens in the plasma of three Japanese macaques that contracted severe thrombocytopenia (ID: HGN174, HGN181 and IZ1470) were examined. Twenty DNA viruses and 13 RNA viruses listed in Table 3 were tested by RT-PCR or PCR based on the previous studies^3^,^12^,^27^,^28^,^29^,^30^,^31^,^32^,^33^,^34^,^35^,^36^,^37^,^38^,^39^,^40^,^41^,^42^,^43^,^44^,^45^,^46^,^47^,^48^,^49^,^50^,^51^,^52^ or using the in-house testing procedures at National Institute of Infectious Diseases, Japan.

### Rapid determination of viral sequences (RDV) method

RDV was performed as described previously^54^,^55^. In this study, RNA and DNA were extracted independently from partially purified viral fractions, which were prepared as described in the “Electron microscopy” section. *Hae* III and *Alu* I restriction enzymes were used to synthesize the second cDNA library.

### Metagenomic analysis

Metagenomic analysis was performed using a high-throughput sequencer. Total RNA was extracted from specimens with TRI-LS (Sigma-Aldrich, Tokyo, Japan) and reverse-transcribed with a Transplex whole transcriptome amplification (WTA1) kit (Sigma-Aldrich)^56^ using a quasi-random primer according to the manufacturer's protocol with modifications (i.e., 70 cycles of PCR). PCR amplification to prepare the template DNA for pyrosequencing was performed using AmpliTaq Gold DNA Polymerase LD (Applied Biosystems, Tokyo, Japan)^56^. Because almost all of the amplified cDNA were within the 200–1,000-bp range, the PCR products were used as templates directly for emulsion PCR in GS Junior pyrosequencing (454 Life Sciences). The obtained data were then subjected to a data analysis pipeline for BLAST search. Data analysis was performed with computational tools using each read sequence, as described previously^56^.

### Detection of SRV-4 viral genome by RT-PCR or PCR

The viral RNAs from Japanese macaques were examined by RT-PCR using oligonucleotide primers that were specifically designed for SRV-4^4^. The partial regions of the *gag* gene were amplified using following two primer sets; aSRV-F1167 and aSRV-R1710, and/or aSRV-F429 and aSRV-R855^4^. RT-PCR was performed with OneStep RT-PCR Kit (Qiagen) as reported previously with some modification^4^. RT-PCR cycles were performed using an iCycler (Bio-Rad, Tokyo, Japan) as follows: 50°C for 30 min, 95°C for 15 min, 40 cycles at 95°C for 30 s, 45°C for 32 s, and 72°C for 75 s, and 1 cycle at 72°C for 2 min^4^.

In addition, the gag region of proviral DNA was amplified by nested PCR using primers (Tga1, Tga2, Tga3, and Tga4) that specifically detected the gag region of the SRV/D-T (SRV-4)^12^.

### Virus isolation

Virus isolation tests with Vero E6 (ATCC CRL-1586), human SLAM-Vero^57^, canine SLAM-Vero cells^58^, and embryonated egg cultures were performed using the plasma or clots from the affected macaques.

PBMCs were isolated from SRV-4 provirus-positive Japanese macaques (ID: TH1626 and IZ1470) by Ficoll gradient centrifugation and washed with PBS. Approximately 5 × 10^5^ PBMCs were cocultured with 2 × 10^5^ Raji cells (a Burkitt's lymphoma B-cell line) in complete medium, which comprised RPMI-1640 supplemented with 10% fetal calf serum, 2-mercaptoethanol (50 μM), sodium pyruvate (1 mM), penicillin (100 IU/ml), streptomycin (100 μg/ml), concanavalin A (10 μg/ml), and recombinant interleukin-2 (200 U/ml). Viral CPE was monitored during cocultivation^5^. DNA from Raji cells cocultured with PBMCs was extracted using a NucleoSpin Blood Kit (Macherey-Nagel, Duren, Germany). Then the *gag* region was amplified by nested PCR using the SRV-4 specific primer set described above^12^.

### Phylogenetic analysis

The partial regions of the *gag* gene were amplified by RT-PCR using the primer set, aSRV-F1167 and aSRV-R1710, as described above. The RT-PCR products were purified using MinElute PCR Purification Kits (Qiagen) or cleaned enzymatically with Calf intestine Alkaline Phosphatase (TOYOBO, Osaka, Japan) and Exonuclease I (TaKaRa, Otsu, Japan). Direct sequencing was performed with a Dye Terminator Cycle Sequencing Kit and an ABI 3130xl generic analyzer (Applied Biosystems). DNA sequences of the samples in this study were combined with previous data (database accession numbers: SIVRV1CG for SRV-1; M16605, AF126468, and AF126467 for SRV-2; M12349 and AF033815 for SRV-3; FJ971077, FJ979638, FJ979639, GQ454446, and AB181392 for SRV-4; and AB611707 for SRV-5) and aligned using the CLUSTALW computer program^59^. Phylogenetic trees were constructed using the neighbor-joining (NJ) method with MEGA 6.0^60^. Evolutionary distances were computed using the maximum composite likelihood method. The phylogenetic tree was evaluated using a bootstrap test based on 1,000 resamplings. The sequence of simian endogenous retrovirus (SERV; STU85505) was used as an outgroup to indicate the location of the root of the ingroup.

## Author Contributions

M.O., T.Miy., S.M., F.O., K.F., T.N., T.Ho., T.Ma. and H.H. conceived and designed the experiments. M.O., T.Y., T.M.-N., J.S., A.W., A.K., R.Y., K.S., T.Miz., N.N., J.T., S.O., M.H., S.N., T.N. and T.I. performed the experiments. S.M., F.O., J.T., T.Miz., S.N., T.N., T.M.-N., J.S. and M.O. analyzed the data. A.W., A.K., A.S., A.M., T.Ha., T.M.-N. and J.S. contributed reagents/materials/analysis tools. M.O., T.Miy., E.S., T.M.-N., H.A. and H.H. wrote the paper.

## Supplementary Material

Supplementary Information Emergence of infectious malignant thrombocytopenia in Japanese macaques (Macaca fuscata ) by SRV-4 after transmission to a novel host

## Figures and Tables

**Table 1:** Hematology of Japanese monkeys that contracted thrombocytopenia

		ID	sex	age	first data at onset				date of death	last data before death				onset to death (days)	remarks
					date of onset	PLT (×10^4^/*μ*l)	WBC (×10^2^/*μ*l)	RBS (×10^4^/*μ*l)		date	PLT (×10^4^/*μ*l)	WBC (×10^2^/*μ*l)	RBC (×10^4^/*μ*l)		
phase1	1	TH1335	♀	11	2001/7/26	1.6	25	345	2001/7/28	2001/7/27	1.6	25	349	2	
	2	NT1374	♀	10	8/6	2.0	24	277	8/8	8/6	1.6	23	281	2	
	3	TH1460	♀	9	2002/4/1	1.9	52	159	2002/6/8	2002/6/7	18.8	92	71	68	blood transfusion
	4	MJ1344	♂	11	4/8	0.3	14	218	4/12	4/8	0.5	13	208	4	
	5	TH640	♀	20	5/1	0.7	20	408	5/22	5/21	43.3	26	141	21	blood transfusion
	6	TH330	♀	26	5/16	1.2	22	474	5/23	5/21	2.5	34	212	7	
	7	TH1495	♀	9	8/1	1.0	75	245	survive	8/16	24.1	45	379	-	*long-term survival
phase2	1	WK1655	♂	12	2008/3/11	0.0	37	225	2008/6/13	2008/6/10	20.7	25	131	94	blood transfusion
	2	TH2158	♂	2	3/14	0.2	98	42	3/15	3/14	0.2	98	42	1	
	3	AR1694	♂	11	5/14	0.2	31	456	5/16	5/14	0.2	31	456	2	
	4	AR1995	♀	5	5/22	0.0	69	109	5/23	-	-	-	-	1	
	5	TH1433	♀	16	12/4	0.6	51	168	12/5	12/4	0.6	51	168	1	
	6	TH2107	♀	2	12/16	0.0	25	172	12/16	12/16	0.0	25	172	0	
	7	AR1988	♂	6	2009/1/13	0.1	-	-	2009/5/8	2009/4/28	19.8	-	-	115	blood transfusion
	8	AR1868	♀	8	4/13	-	-	-	4/13	-	-	-	-	0	
	9	TH1863	♀	9	5/19	0.0	8	363	5/19	5/19	0.0	8	363	0	
	10	TH1735	♀	11	6/17	0.4	39	138	survive	7/3	18.1	62	189	-	*long-term survival
	11	TH2041	♂	5	6/19	0.0	34	190	6/22	6/22	0.0	22	86	3	
	12	AR1797	♀	10	7/19	-	-	-	7/19	-	-	-	-	0	
	13	AR1743	♀	11	7/27	0.0	35	189	7/27	7/27	0.0	35	189	0	
	14	AR1856	♀	9	8/17	-	-	-	8/17	-	-	-	-	0	
	15	WK1649	♂	13	9/4	-	-	-	9/5	-	-	-	-	1	
	16	HGN170	♀	4	9/9	0.2	87	96	9/10	9/9	0.2	87	96	1	
	17	TH1861	♀	9	9/16	0.1	55	366	9/20	9/16	0.1	55	366	4	
	18	US2026	♀	8	11/19	0.3	10	319	11/20	11/20	0.0	6	270	1	euthanasia
	19	OK2015	♂	10	12/8	0.6	40	411	12/17	12/14	39.8	22	373	9	
	20	AR2182	♂	2	12/9	5.1	94	391	12/22	12/22	4.6	54	277	13	euthanasia
	21	HGN266	♂	0	12/8	-	-	-	12/8	-	-	-	-	0	
	22	MNN326	♂	0	12/14	0.0	93	88	12/15	12/14	0.0	93	88	1	
	23	TH1439	♂	18	2010/1/6	4.6	20	529	2010/1/8	2010/1/8	1.5	11	409	2	euthanasia
	24	HGN189	♀	1	1/29	1.2	132	172	2/1	1/29	1.2	132	172	3	
	25	HGN174	♀	adult	2/5	0.9	52	166	2/5	2/5	0.9	52	166	0	euthanasia
	26	HGN159	♀	adult	2/8	-	-	-	2/8	-	-	-	-	0	
	27	HGN176	♀	adult	2/8	-	-	-	2/8	-	-	-	-	0	
	28	HGN181	♀	6	2/15	0.3	82	187	2/16	2/15	0.4	76	161	1	euthanasia
	29	HGN157	♀	adult	2/15	0.4	18	471	2/17	2/15	0.4	18	471	2	euthanasia
	30	HGN160	♀	adult	2/15	7.3	135	562	2/18	2/17	18.8	120	462	3	
	31	HGN155	♀	adult	3/1	1.6	51	159	3/3	3/3	0.4	18	488	2	euthanasia
	32	HGN156	♂	adult	3/15	0.0	51	370	3/16	3/16	1.1	39	302	1	euthanasia
	33	HGN267	♂	1	2/15	8.5	143	459	3/17	3/17	13.4	118	538	2	euthanasia
	34	HGN165	♀	5	3/10	9.2	87	277	3/31	3/31	17.6	49	268	21	euthanasia
	35	HGN161	♀	adult	4/5	0.0	4	282	4/5	4/5	0.0	4	282	0	euthanasia
	36	TH1847	♀	10	4/7	0.3	5	369	4/7	4/7	0.3	5	369	0	
	37	TH1949	♂	8	4/7	0.0	34	298	4/7	4/7	0.0	34	298	0	
	38	TH1626	♀	14	5/7	0.2	15	341	5/8	5/7	0.2	15	341	1	euthanasia
	39	IZ1470	♂	17	6/7	3.3	36	488	6/11	6/11	0.1	-	-	4	euthanasia
	40	TBN201	♀	6	7/20	0.3	23	401	7/20	7/20	0.3	23	401	0	euthanasia
	41	HGN172	♀	adult	9/8	0.0	14	304	9/8	9/8	0,0	14	304	0	euthanasia
	42	HGN167	♀	adult	12/7	1.4	29	94	12/17	12/17	1.3	14	146	10	euthanasia,blood transfusion
	43	MRN418	♀	1	2011/2/17	-	-	-	2011/2/17	-	-	-	-	0	

The hyphen (-) indicates that data was not obtained.

**Table 2:** Detection of antibodies against simian viral agents

Virus	BV	CMV	SEBV	SFV	SIV	SRV	STLV	SVV
Deaths by thrombocytopenia n = 14 (%)	5 (35.7)	14 (100)	13 (92.9)	14 (100)	0 (0)	0 (0)	3 (21.4)	0 (0)
Healthy control (2nd campus) n = 10 (%)	2 (20)	10 (100)	10 (100)	10 (100)	0 (0)	0 (0)	5 (50)	0 (0)

BV: B virus, CMV: simian cytomegalovirus, SEBV: simian Epstein–Barr virus, SFV: simian foamy virus, SIV: simian immunodeficiency virus, SRV: simian retrovirus, STLV: simian T-lymphotropic virus, SVV: simian varicella virus.

**Table 3:** List of viruses tested by PCR or RT-PCR

	DNA virus	reference		RNA virus	reference
1	Hepatitis B Virus	[27]	1	Filovirus consensus	[42]
2	Papillomavirus 16/18/31/52 and consensus	[28, 29]	2	Reston ebolavirus	in-house
3	Human bocavirus	[30]	3	Simian hemorrhagic fever virus	[43]
4	Adenovirus	[31]	4	Rhinovirus A and B	[44]
5	Herpes simplex virus1/2	[32]	5	Rhinovirus C	[45]
6	Varicella zoster virus	[32]	6	Parechovirus	[46]
7	Epstein–Barr virus	[32]	7	Coronavirus consensus	[47, 48, 49]
8	CMV cytomegalovirus	[32]	8	Parainfluenza virus 1, 2, 3	[44]
9	Human herpesvirus 6	[33]	9	Reovirus consensus	[50]
10	Human herpesvirus 7	[33]	10	Flavivirus consensus	[51]
11	Human herpesvirus 8	[34]	11	Picornavirus consensus	[52]
12	Transfusion transmitted virus	[35]	12	SRV (Simian type D retrovirus)	[3, 53]
13	JC virus	[36]	13	SRV-4 (Simian type D retrovirus 4)	[12]
14	Adeno-associated virus 2,3	[37]			
15	Parvovirus B19	[38]			
16	Parvovirus (Erythrovirus consensus)	in-house			
17	Simian Parvovirus	in-house			
18	Polyomavirus consensus	[39]			
19	Adenovirus consensus	[40]			
20	Herpesvirus consensus	[41]			

in-house: the in-house testing procedures at National Institute of Infectious Diseases, Japan.
