# Supplementary material for: Emergence of infectious malignant thrombocytopenia in Japanese macaques (Macaca fuscata) by SRV-4 after transmission to a novel host
Source: Sci Rep. 2015 Mar 6;5:8850. doi: 10.1038/srep08850 (PMC4351523; doi:10.1038/srep08850)

Supplementary Information

Emergence of infectious malignant thrombocytopenia in Japanese macaques (*Macaca fuscata*) by SRV-4 after transmission to a novel host

Munehiro Okamoto[1*], Takayuki Miyazawa[2], Shigeru Morikawa[3], Fumiko Ono[4], Shota Nakamura[5], Eiji Sato[1], Tomoyuki Yoshida[1], Rokusuke Yoshikawa[2], Kouji Sakai[3], Tetsuya Mizutani[3], Noriyo Nagata[3], Jun-ichiro Takano[4,6], Sachi Okabayashi[4], Masataka Hamano[4], Koji Fujimoto[4], Takaaki Nakaya[5,7], Testuya Iida[5], Toshihiro Horii[5,8], Takako Miyabe-Nishiwaki[1], Akino Watanabe[1], Akihisa Kaneko[1], Akatsuki Saito[1], Atsushi Matsui[1], Toshiyuki Hayakawa[1], Juri Suzuki[1], Hirofumi Akari[1], Tetsuro Matsuzawa[9] & Hirohisa Hirai[10]

[1]Center for Human Evolution Modeling Research, Primate Research Institute, Kyoto University, Inuyama, Aichi 484-8506, Japan
[2]Laboratory of Signal Transduction, Department of Cell Biology, Institute for Virus Research, Kyoto University, Kyoto 606-8507, Japan.
[3]National Institute of Infectious Diseases, Toyama 1-23-1, Shinjuku-ku, Tokyo 162-8640, Japan
[4]The Corporation for Production and Research of Laboratory Primates, 1-16-2 Sakura, Tsukuba, Ibaraki 305-0003, Japan
[5]Department of Infection Metagenomics, Genome Information Research Center, Research Institute for Microbial Diseases, Osaka University, 3-1 Yamadaoka, Suita, Osaka 565-0871, Japan
[6]Tsukuba Primate Research Center, National Institute of Biomedical Innovation, 1-1 Hachimandai Tsukuba, Ibaraki 305-0843, Japan
[7]Department of Infectious Diseases, Kyoto Prefectural University of Medicine, 465 Kawaramachi-hirokoji, Kamigyo-ku, Kyoto, Japan
[8]Department of Molecular Protozoology, Research Institute for Microbial Diseases, Osaka University, Suita, Osaka, Japan
[9]Department of Brain and Behavioral Sciences, Primate Research Institute, Kyoto University, Inuyama, Aichi 484-8506, Japan
[10]Department of Molecular Biology, Primate Research Institute, Kyoto

University, Inuyama, Aichi 484-8506, Japan

Supplementary Figure Legends:

Supplementary Figure S1. Metagenomic detection of SRV-4 in plasma of a Japanese macaque. 485 detected sequence reads were mapped onto the reference genome of SRV-4 (GenBank ID: FJ971077). the upper box represents the SRV-4 genome. Sequence reads detected are shown as bars colored by homology (% identity). Detected positions are colored in the box of the genome cumulatively.

Fig. S1

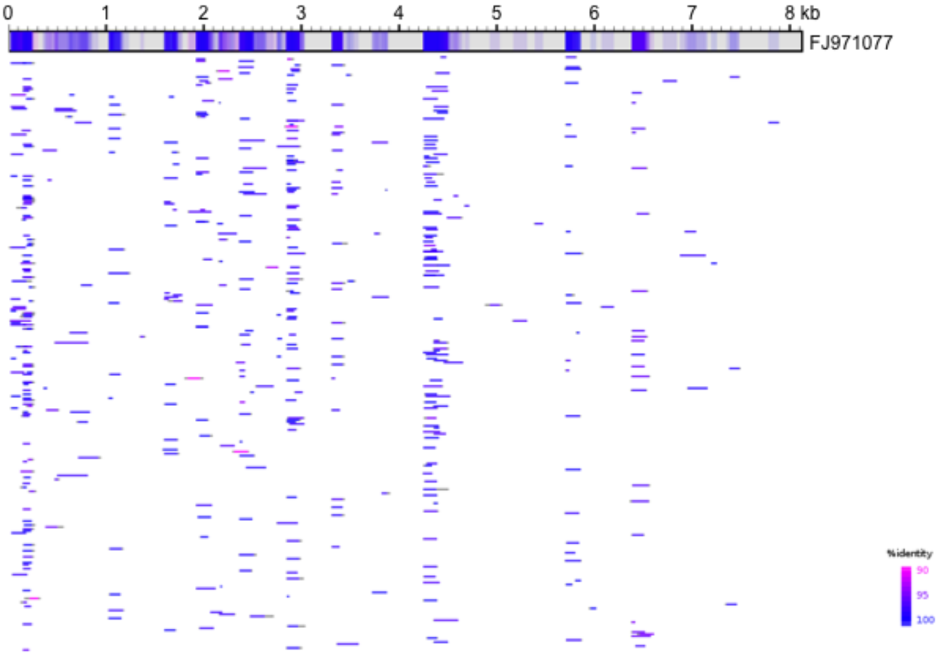

Supplement: Supplementary Information — Emergence of infectious malignant thrombocytopenia in Japanese macaques (Macaca fuscata ) by SRV-4 after transmission to a novel host [file srep08850-s1.pdf]
